# Association between Fever and Primary Tooth Eruption: A Systematic Review and Meta-analysis

**DOI:** 10.5005/jp-journals-10005-1453

**Published:** 2017-02-27

**Authors:** Mariana A Nemezio, Katharina MH De Oliveira, Priscilla C Romualdo, Alexandra M Queiroz, Francisco WG Paula-e-Silva, Raquel AB Silva, Erika C Küchler

**Affiliations:** 1 Assistant Professor, Department of Dentistry, Tiradentes University, Maceio, Alagoas Brazil; 2 Assistant Professor, Department of Dentistry, Federal University of Sergipe, Lagarto Sergipe, Brazil; 3 Postdoctoral Researcher, Department of Pediatric Dentistry, University of Sao Paulo Ribeirao Preto, Sao Paulo, Brazil; 4 Associate Professor, Department of Pediatric Dentistry, University of Sao Paulo Ribeirao Preto, Sao Paulo, Brazil; 5 Research Associate, Department of Pediatric Dentistry, University of Sao Paulo Ribeirao Preto, Sao Paulo, Brazil; 6 Associate Professor, Department of Pediatric Dentistry, University of Sao Paulo Ribeirao Preto, Sao Paulo, Brazil; 7 Research Associate, Department of Pediatric Dentistry, University of Sao Paulo Ribeirao Preto, Sao Paulo, Brazil

**Keywords:** Fever, Primary tooth, Tooth eruption.

## Abstract

**Aim:**

To perform a systematic review and meta-analysis to establish if fever is associated with primary tooth eruption.

**Materials and methods:**

Literature searches involved Pubmed, MEDLINE, Web of Science, Scopus and Cochrane. The potentially relevant studies had the full text analyzed. Only studies concerning fever during eruption period of primary tooth in humans were included. Papers in non-English language, and papers that included syndromic patients or patients with any disease were excluded. The meta-analyses were performed with Review Manager (version 5.3). Only studies that reported the results as dichotomous data were analyzed with Cochran-Mantel-Haenszel test in meta-analysis function of Review Manager 5.3. The fixed-effects model was used to evaluate the association between tooth eruption and fever.

**Results:**

Search identified 83 potential studies. After exclusion of the duplicated studies, or were not related to the criteria of inclusion only 6 studies were selected for the systematic review. In the overall meta-analysis, no association was found [OR = 1.32 (0.88-1.96)] between fever and primary tooth eruption. However, in the subgroup analysis, when the method used to measure fever was the rectal temperature there was an association [OR = 2.82 (1.55-5.14)] between fever and primary tooth eruption.

**Conclusion:**

There are few suitable studies in the literature regarding the association between primary tooth eruption and fever. However, our study found an association between fever and primary tooth eruption only when rectal temperature was performed

**How to cite this article:**

Nemezio MA, De Oliveira KMH, Romualdo PC, Queiroz AM, Paula-e-Silva FWG, Silva RAB, Kuchler EC. Association between Fever and Primary Tooth Eruption: A Systematic Review and Meta-analysis. Int J Clin Pediatr Dent 2017;10(3):293-298.

## BACKGROUND

Tooth eruption is a natural physiological process that consists of the tooth migrating from its intraosseous position in the jaw to appear in the oral cavity. It involves many physiological mechanisms.^1^ The eruption of primary tooth usually begins from 4 to10 months after birth. The full primary dentition with twenty primary teeth is almost always completed in a 30-months-old.^2^ The process of tooth eruption is under strong genetic control with minor influence from environmental factors.^3^

Primary tooth eruption has been associated with some symptoms that include irritability, gingival irritation, increased salivation, restless sleep, diarrhea, loss of appetite, and fever.^4-9^ Among these symptoms, fever is the most frequently reported by mothers^7,8,10-13^ and health care professionals.^6,14,15^

Fever is defined as body temperature above the normal of 98.6°F (37°C). The body temperature measurement is most commonly taken to confirm the presence or absence of fever and it is an important physical sign in many childhood diseases. Many decisions concerning the investigation and treatment of children are based on the results of temperature measurement. To determine the presence of fever in young children is particularly important in order to detect the illness.^16-20^

The issue of symptoms associated with tooth eruption has been controversial; some studies reported that specific symptoms are associated with tooth eruption, while others failed to demonstrated this association.^7,11,20-22^ This is particularly true in the case of fever. Thus, the aim of this study is to perform a systematic review and meta-analysis to establish if fever is associated with primary tooth eruption.

## MATERIALS AND METHODS

This review was based on Prisma Statement (www. prismastatement.org). The concept of the study was first registered in the international prospective register of systematic reviews, "PROSPERO" (CRD42015019994).

### Search Strategy

The search strategy was based on the following Medical Subject Heading (MeSH) terms or Text Word [tw] in different combination strategies: "Tooth eruption" [MeSH terms] or "Dental eruption" [tw] or "Primary teeth eruption" [tw] or "Deciduous tooth eruption" [tw] and "Fever" [MeSH terms].

The literature searches involved PubMed MEDLINE, Web of Science, Scopus, and Cochrane. The potentially relevant studies had their full texts analyzed and were included since they met all the exclusion criteria in the systematic review.

Two examiners (MAN and KM) evaluated titles, abstracts, and full text and if there was a diverging opinion, the disagreement among examiners was reexam-ined in consensus meetings with a third examiner (PCR).

### Eligibility Criteria and Outcome Measures

Only studies concerning fever during eruption period of primary tooth in humans were included. Papers in non-English language and papers that included syn-dromic patients or patients with any systemic disease were excluded.

The review design is presented in Table 1, following the PECO strategy. Only the studies that followed the PECO criteria were included. All studies should measure the body temperature at least once during the primary tooth eruption and at a time that was not associated with tooth eruption.

An assessment of quality and risk of bias of the studies included in the systematic review was performed. The checklist included questions on the study design, sample size calculation, inclusion and exclusion criteria, and statistical analyses. When evaluating the criteria for each study, the reviewers (ECK and MAN) assigned problems for each criterion as low, high, or unclear in terms of their expected effect on the results. The decision was made as to whether the methods were adequate for producing high-quality information.

The meta-analyses were performed using the Review Manager software (version 5.3). Forest plots and summary risk of bias were created with this software. At this stage, only studies that reported the results as dichotomous data were analyzed with Cochran-Mantel-Haenszel test in meta-analysis function of Review Manager 5.3. The fixed-effects model was used to evaluate the association between tooth eruption and fever. The pooled odds ratio was used to compare the relative odds of the fever during tooth eruption (confidence interval = 95%).

The I^2^ statistic was used to assess statistical heterogeneity between studies, where I^2^ values of 25, 50, and 75% indicated low, medium, and high heterogeneity respectively.

**Table 1:** **Table 1:** Description of the population exposure comparison and outcome

*Acronym*		*Description*	
P (population)		Toddler	
E (exposition)		Primary tooth eruption	
C (comparison)		Noneruption and during eruption period	
O (outcome)		Fever	

## REVIEW RESULTS

### Study Identification and Characteristics

Flow Chart 1 shows a flow diagram describing the process of studies selection related to fever and primary tooth eruption. Search (until March 2016) at PubMed MEDLINE identified 56 potential studies, the Web of Science search identified 5 potential studies, the Scopus search identified 20, and the Cochrane search identified 2 studies.

After exclusion of the duplicated studies (7), the remaining were analyzed and excluded if the subjects were not related to the proposed by title reading (60) or abstract reading (5). Thus, 56 were excluded because they were not related to the subject, 4 because were in other languages, 1 because it was a review study and 4 because they were case reports.

Eleven studies were followed for full-text analysis. Five studies did not measure fever and evaluated tooth eruption or were based on the parents beliefs or reports and, therefore, were excluded. Thereby, six studies were selected for the systematic review. A summary of the selected was described in Table 2.

### Quality Assessment and Risk of Bias

**Fig. 1:**
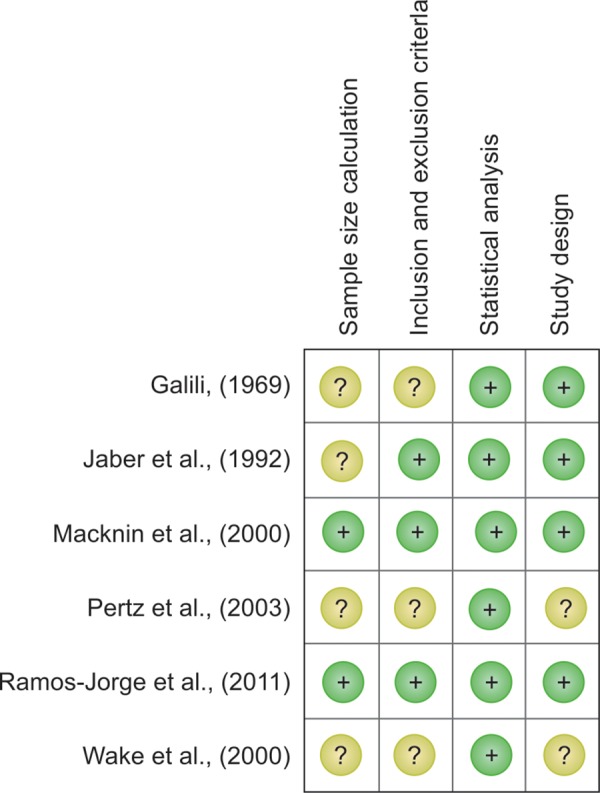
Quality assessment of the included studies (The Cochrane Collaboration tool for assessing risk of bias)

The quality assessment of the prospective longitudinal studies revealed generally proper reporting and the presence of low or unclear risk of bias (Fig. 1). One case-control study presented a high risk of bias.^8^ Of those, only 2 studies calculated sample size.^7,10^ In two studies, the inclusion and exclusion criteria were satisfactorily described.^7,10^ In all studies, the statistical analysis was satisfactory.

### Overall Summary

The studies performed by Ramos-Jorge et al^10^ and Wake et al^11^ were not included in the meta-analyses due the fact that their results were presented as a continuous variable and were unable to be dichotomized. They were also not comparable.

**Fig. 2:**
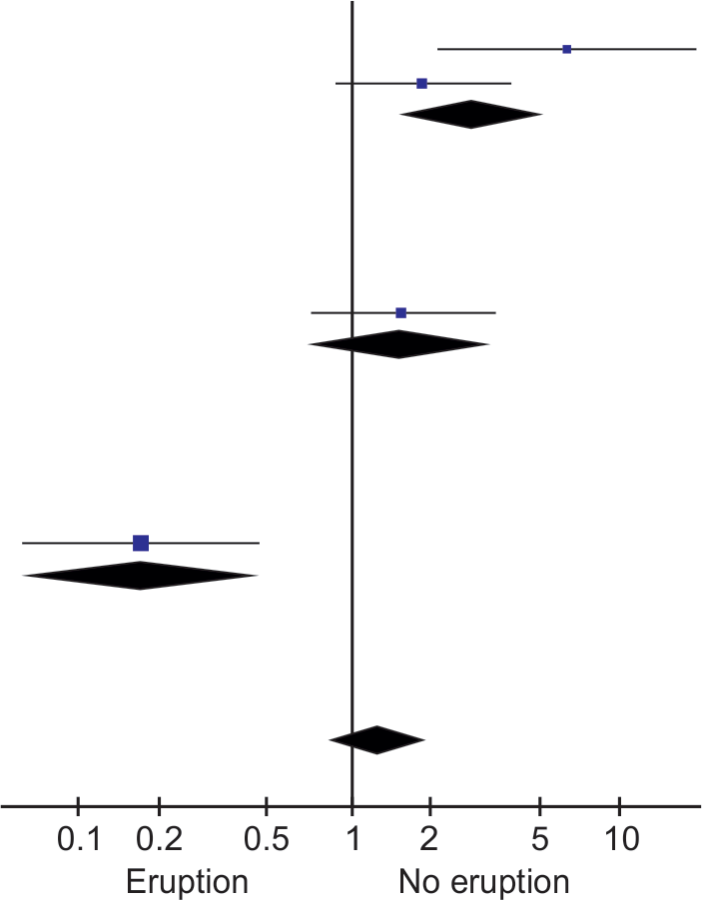
Forest plot

Figure 2 presents the Forest plot for the meta-analysis. In the subgroup analysis, according to the site at the body used to record the temperature, the rectal method presented an association between fever and primary tooth eruption (OR = 2.82 [1.55-5.14]). In the overall meta-analyses results, fever was not associated with primary tooth eruption (OR = 1.32 [0.88-1.96]). Substantial quantitative heterogeneity was found on the multiplicative scale (I^2^ = 88%, p < 0.0001).

**Flow Chart 1:**
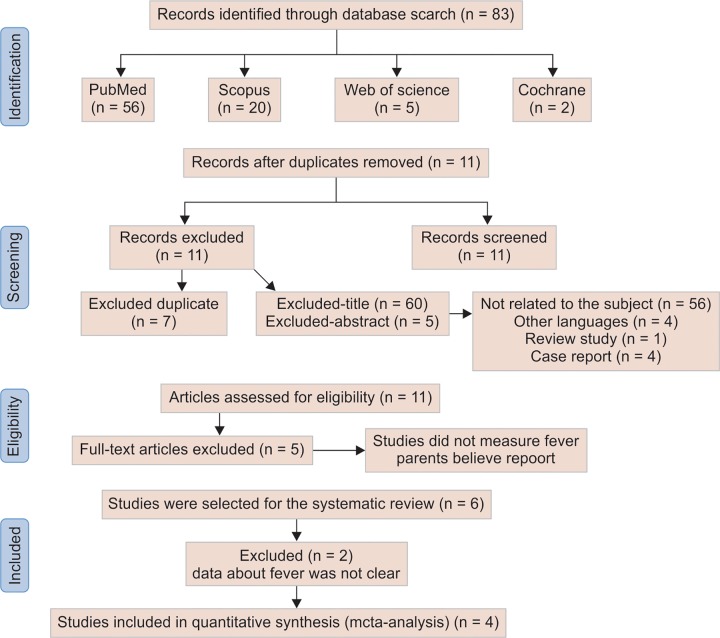
Stages of the study selection progression according to Prisma Statement

## DISCUSSION

Previous studies have attempted to evaluate body temperature alteration as a sign associated with primary tooth eruption. However, this issue is still unclear in pediatric research. Although many studies reported this association, our meta-analyses were not able to confirm this. Our results could probably be influenced by the heterogeneity among the studies included in the meta-analyses.

Different sites on the body can be used to record the body temperature. In addition, different thermometers, such as mercury-in-glass and liquid-in-glass, electronic with digital display, infrared or tympanic, contact or noncontact temporal artery thermometer, or disposable chemical thermometers are devices commonly used in young children.^18^ The included studies had different methods to detect fever.

In studies that used the rectal temperature, there was an association between fever and tooth eruption^12,13^ and our meta-analysis of the pooled results of these two studies confirmed that fever is almost three times more common in the primary eruption period. The studies that used axillary and tympanic temperatures also found an association between fever and tooth eruption.^10^ Interestingly, the three studies, in which the type of method to detect fever was not clear,^8^ did not find association between these two conditions. In fact, previous studies showed that measurement of temperature by ear thermometer was less accurate than rectal temperature.^23-25^ This may explain our meta-analysis results.

Many studies in the literature attempting to compare rectal, oral, tympanic, and axillary thermometers have been conducted to find the most appropriate thermometer and the best anatomical site for temperature mea-surement.^16,26-31^ Axillary thermometry is less invasive, but does not reflect core temperature and it is largely influenced by ambient temperature and vasoactivity.^26^ Rectal temperature is about 1°F (0.5°C) higher than an oral temperature and 2°F (1°C) higher than an axillary temperature.^32^

Another concern that may be considered is the subject who performed the temperature measurements. It has been reported in literature that readings of temperature obtained by parents differ from those obtained by a nurse using the same instrument by a clinically significant amount (0.5°C or more).^33^ The sample selection can also influence the outcome. Some studies investigated samples with low risk of bias.^34,35^ In addition, the outcome assessment was different according to the study design and might also influence the final result of this meta-analyses.

**Table 2:** **Table 2:** Overview data extracted from included studyr

*Authors*		*Sample characteristics (sample size and age range in months)*		*Study design*		*Tooth eruption and fever evaluation*		*Fever* *measurement*		*Description of the outcome assessment*		*Comments and/or limitations*		*Conclusions*	
Ramos-Jorge etal^10^		(n = 47), Age range: 5-15		Prospective longitudinal Paired evaluation		Trained dentists evaluated tooth eruption and body temperature		Axillary and tympanic thermometers. Fever was recorded as continuous variable		Data collection began before the eruption of at least one incisor and ended 1 week after the eruption of the last incisor		In order to minimize the variation, the tooth eruption and the temperature evaluations were performed at the same time. Noninstitutionalized infants were used in order to reduce the bias of viral and bacterial infection dissemination in daycares. The exact periods of the temperature measurement were unclear		During eruption days children presented higher temperatures (p<0.01)	
Peretz et al^8^		Eruption group, (n = 340), No eruption group, (n = 145), Age range: 4-36		Case-control		Nurse evaluated the temperature. The person that evaluated the tooth eruption was unclear		The thermometer used was unclear. Fever was recorded as a dichotomous data		During the eruption of the incisors, fever was recorded		Did not report the fever as a continuous variable and did not report the type of the thermometer. Convenience sample from the Pediatric Clinic was used. The time of the evaluation was unclear		There was no association between fever and tooth eruption (p > 0.05)	
Wake etal^11^		(n = 21), Age range: 6-24		Prospective longitudinal Paired evaluation		Dental therapist evaluated tooth eruption and temperature		Tympanic thermometers. Fever was recorded as dichotomous and continuous variables		Analyses: 1st defining a toothday as any of the 5 days surrounding the eruption and 2nd, defining a toothday as any of the 5 days leading up to and including the eruption day		A convenience sample of children from a care center was used. Statistical analyses were not reported		There was no association between fever and tooth eruption (p > 0.05)	
Macknin et al^7^		(n = 111), Age range: 3-5.6		Prospective longitudinal Paired evaluation		Trained parents by pediatrician nurse		Tympanic thermometers. Fever was recorded as dichotomous variables		Parents were told to take measurements before recording the highest temperature reading. They should record the temperature twice a day (morning and evening)		A convenience sample of children from clinic-based group pediatric. Fever characterization and the evaluation of tooth eruption were not described		Tooth eruption was associated with slight temperature elevation (p > 0.05)	
Jaber et al^12^		(n = 46), Age range: 6-18		Prospective longitudinal Paired evaluation		Mothers and health care professional		Rectal temperature. Fever was recorded as a dichotomous data		Mothers recorded the temperature and were told to bring the baby for professional evaluation of the tooth eruption when it was suspected		The parents performed the fever analyses and did not receive a previous training		There was an association between fever and tooth eruption (p< 0.025)	
Galili etal^13^		(n = 43), Age range: 5-23		Prospective longitudinal Paired evaluation		Nurse evaluated the temperature. Dentist evaluated the tooth eruption		Rectal temperature. Fever was recorded as a dichotomous data		7 days prior to the tooth eruption was designated as "tooth eruption"		A convenience sample of children from "baby home"		There was an association between fever and tooth eruption (p<0.05)	

Only three studies fully described the inclusion and exclusion criteria. They attempted to report the characteristics and health condition of the included children.^7,10,11^ The other 3 studies only reported gender and age.^8,12,13^

## CONCLUSION

In conclusion, there are few suitable studies in the literature regarding the association between primary tooth eruption and fever. However, our study found an association between fever and primary tooth eruption only when rectal temperature was performed. Therefore, we believe that further studies should be conducted to shed light on this relationship using different methods for measurement of body temperature.

## CLINICAL SIGNIFICANCE

Primary tooth eruption has been associated with several symptoms, and fever is the most frequent one reported by mothers and health care professionals. The issue of symptoms during tooth eruption is controversial and it is very important not to underestimate the presence of fever during teething, ensuring that it is not due to other infections.
